# ‘I went there in an effort to help the child, but you can see there is corruption in the world’: Adults’ conceptualisations and enactments of child protection in schools in a challenging context

**DOI:** 10.1371/journal.pone.0275918

**Published:** 2022-10-20

**Authors:** Ellen Turner, Robert Nyakuwa, Tendai Nhenga-Chakarisa, Charles Muchemwa Nherera, Annah Theresa Nyadombo, Dorcas Mgugu, Caroline Trigg, Camilla Fabbri, Sarah Rank, Karen Devries

**Affiliations:** 1 Faculty of Public Health and Policy, Department of Global Health and Development, London School of Hygiene and Tropical Medicine, London, United Kingdom; 2 Q Partnership, Harare, Zimbabwe; 3 Child Rights Research Centre, Africa University, Mutare, Zimbabwe; 4 Department of Art and Design Technology Education, University of Zimbabwe, Harare, Zimbabwe; 5 Education Commission, Zimbabwe Catholic Bishops Conference, Harare, Zimbabwe; 6 Porticus Africa, Nairobi, Kenya; Washington University in St. Louis, UNITED STATES

## Abstract

Schools have the potential to be sites of support for vulnerable children, but can also be sites of violence perpetration. In this qualitative study we explore how adult school stakeholders in and around two public Catholic primary schools in Zimbabwe conceptualise and enact child protection. We analysed our findings in light of the protracted economic crisis in Zimbabwe; the current policy context for child protection; and the Covid-19 pandemic. We conducted semi-structured interviews with 18 adult education stakeholders in and around one rural and one urban school in the Harare Archdiocese, from October 2020 to January 2021. This comprised 12 school-level stakeholders, including teachers (N = 4), parents (N = 4), school priests (N = 2), and headteachers (N = 2), who were the main focus of this study, and a further 6 high-level education actors. We employed thematic analysis. Adults in this study placed considerable responsibility on children to protect themselves, with often unreasonably high expectations of children’s capacity to prevent abuse. At times they also blamed and stigmatised children, which was gendered, and particularly emerged around adolescent sexuality. Our findings suggest that this was linked to social norms around discipline, protection and gender, but in particular, the way these emerged in relation to the challenges of the context. Policy and interventions to promote child protection in schools in Zimbabwe should incorporate both an attention to the challenges teachers face in contexts of adversity, as well as address a tendency for adult school stakeholders to hold children responsible for their own protection.

## Background

Violence against children, including child marriage, has been declared a world health emergency [1] and commitments to reduce violence feature explicitly in Sustainable Development Goals [SDGs] 4 (education), 5 (gender equality) and 16 (peace and justice). Children spend more time in school than anywhere else outside the home [2], meaning that schools have the potential to play a primary role in both the prevention of and response to child protection risks. However, schools themselves can be sites of risk, as both teachers and peers are common perpetrators of physical, emotional and sexual abuse [3–5].

In sub-Saharan Africa, the potential for schools to be sites of care and support for children has largely been explored through their role in responding to HIV and AIDS [6–11]. International policy focus on school care of vulnerable children, emphasising emotional support [8], has been critiqued for not taking account of schools’ realities in resource-poor environments [6, 12–14]. Discrepancies also exist between policy approaches to child protection and teachers’ perspectives; for example in Zimbabwe where teachers prioritised discipline and material support as important forms of care, over emotional support [7]. Also in Zimbabwe, amid contexts of adversity where teachers themselves face unresolved challenges, teachers can feel overburdened, demoralised and even resentful of children [7, 9], and that their own painful experiences are overlooked [6]. Some studies in sub-Saharan African settings show the potential of schools as sites for disclosure and referral to social and health services [10, 15, 16], or as sites for violence prevention interventions [15, 17], however, also highlighting the support, resources and improvements to many schools’ everyday functioning needed to operationalise this [10, 13, 14]. Sociological studies of education also emphasise how schools are sites wherein gendered norms, inequalities and identities can be both formed and reinforced, and that this shapes teachers’ interactions with children around violence, as examined in sub-Saharan Africa [18–22], and in Zimbabwe [23, 24]. Few studies examine teachers’ notions of care within child protection, while also taking account of the role teachers can play in perpetrating violence. Similarly, little is known about how key education actors and stakeholders conceptualise child protection is conceptualised as part of schools’ and teachers’ roles in challenging contexts, and the gendered dimensions of this.

This study draws on qualitative data collected in and around two public Catholic primary schools facing many contextual challenges in Zimbabwe, to examine how adult school stakeholders, including teachers, parents, headteachers and priests, conceptualise and enact child protection. The National Case Management System [NCMS] for children’s welfare and protection in Zimbabwe defines child protection as, ‘a set of services and mechanisms put in place to prevent and respond to violence, abuse, exploitation and neglect, which threaten the well-being of children’ [25]. This paper first explores how adult stakeholders conceptualised the notion of child protection. Secondly it examines school staff descriptions of how child protection concerns were viewed, handled and responded to, using the term ‘enactment’ to encompass the range of actors and interpretations that policy action in schools entails [26, 27]. Finally, we consider how these engagements with child protection related to contextual challenges. The paper concludes with implications of these findings for policy and interventions to prevent and respond to violence in schools in challenging contexts.

### School contexts in Zimbabwe

Zimbabwe has faced two decades of protracted economic challenges, exacerbated by the Covid-19 pandemic. There are routine shortages of clean water, electricity, and fuel. In 2019, 34% of Zimbabweans were living in extreme poverty [28], rising to almost half the population by 2020 [29]. Inflation and currency shifts have impacted teachers’ pay, with basic monthly salaries dropping from an average of 520–550 USD in 2018 to 100–250 USD in 2020–2021, amid escalating costs of living. The situation is unstable and is continuing to worsen to the present day, amid ongoing inflation [30]. In addition, schools have been sites of political intimidation [31, 32]. Challenging conditions and lowering social status have led to feelings of demoralisation and disempowerment among teachers [9, 33, 34]. In May 2021, a federation of teachers’ unions released a report into disturbing conditions in schools, highlighting that many lacked water supply; had overcrowded classrooms; were underprepared to reopen amid the Covid-19 pandemic; and describing physical assaults on teachers from community members, leading to widespread fear and insecurity [35]. These conditions have led to strikes and ongoing tensions between teachers and government. Despite challenges of fragmentation and limited power to affect government policy, teachers’ unions have a strong presence in Zimbabwe with wide national coverage and a strong membership base. All nine unions released a joint statement declaring teachers’ ‘incapacitation’ in September 2020 [36], referring to the acknowledgment that teachers are unable to perform their duties due to lack of adequate remuneration, and encouraging teachers to support this move. Unions urged their members to attend school 1–2 days a week, commensurate with their salaries [37]. This has led to low and patchy teacher attendance across the country, with unions in 2022 estimating that 90% of public school teachers were at least partially out of school [38], and heightened low levels of teacher motivation [34, 39].

There are 124 Catholic primary schools in Zimbabwe, overseen by the Education Commission of the Zimbabwe Catholic Bishops Conference (ZCBC). Roughly one fifth of Catholic schools are owned and run by religious congregations, such as Jesuit or Dominican orders. A small proportion of these congregational schools are private schools, in which teachers’ salaries are paid by the religious congregations. In the vast majority of Catholic schools the school management is Catholic, but teachers’ salaries are paid by the government and many teachers and pupils are not Catholic. Therefore Catholic public schools face many of the same challenges as government public schools, although tend to be better resourced.

### Child protection in Zimbabwe

Over a quarter of children in Zimbabwe experience physical, sexual or emotional violence in childhood [40]. Physical violence is the most common form [40], underpinned by child-rearing norms that support physical punishment [41–43], which children experience most often from caregivers and also from teachers [40]. Sexual violence also occurs, with 9% of girls and 1% of boys experiencing contact sexual violence, from a range of perpetrators including intimate partners, family members, adult community members and school peers [40]. Child marriage is common, with nearly 5% of women being married before the age of 15, and many children engage in labour [41]. It is not clear, however, how far schools are in a position to prevent and/or respond to these forms of abuse.

Zimbabwe has extensive policy and legal frameworks to address these multi-faceted risks to children’s protection, but there are gaps in implementation. Corporal punishment was until recently legal in schools in certain circumstances, for example when punishment was deemed to be ‘moderate’, only by a senior teacher, and to male pupils only [44]. It was fully prohibited in schools in the Education Amendment Act of 2020. This Act does not stipulate repercussions for perpetrators, however, with the onus placed on responsible authorities managing schools (e.g. the Catholic Church) to develop regulations. It is unclear how far this has been done across the country, or how well communicated these policy shifts have been to school staff. Therefore while teachers are common perpetrators of physical violence against children [40], cases are rarely reported. Policy frameworks likewise exist for preventing and responding to child sexual abuse, including laws that prohibit sexual relations with children under 16 years (Criminal Law (Codification and Reform) Act, 2004), and that require mandatory reporting of child sexual abuse (Children’s Act, 2001). These policies are, however, poorly enforced and response mechanisms are weak, with challenges including resource constraints [45]; contradictions or lack of clarity in policies; and conflicts between legal frameworks and social norms, as seen in qualitative evidence from Victim-Friendly Court professionals [46]. While qualitative evidence suggests that teachers can be perpetrators of sexual abuse in Zimbabwe [47, 48], and may be more common than large-scale estimates suggest [40], these cases are therefore similarly rarely reported.

Responding to child welfare concerns is enshrined in law in Zimbabwe through the Children’s Act. The 2017 NCMS, led by the Ministry of Public Service, Labour and Social Welfare, stipulates structures for responding to abuse in the community, but maps inconsistently onto schools’ structures. It requires teachers to ‘[report] any concerns about signs of potential abuse, neglect or other indications of need to the designated teacher for child protection’ [25]. There is limited guidance, training or support for designated child protection teachers, and ultimately decisions on how to handle cases are often left to schools themselves. There is no national policy for child protection specifically in schools. ZCBC has developed a child protection policy for Catholic schools, currently being formulated into an intervention to support its enactment.

## Methods

### Study setting

This study was conducted in two public Catholic primary schools in low-income areas within the Harare Archdiocese, one a high-density urban residential area in Harare and one in a rural setting 100 kilometres outside Harare. The main livelihoods are street vending for the urban school community, and subsistence farming for the rural school community. The rural school is close to a mining area, with some parents and children, and at times teachers, working in the mines. The urban school had roughly 920 pupils enrolled and 34 teachers, while the rural school had roughly 477 pupils enrolled and 13 teachers. Both schools had recently reopened to exams classes only, following Covid-19 school closures. Teachers in both schools were affected by incapacitation, meaning that teacher attendance at school was patchy, many teachers had taken on other work alongside teaching, and levels of motivation were low. Our data showed that adults in and around both schools were concerned about a rise in drug and alcohol abuse and early pregnancy among children during school lockdowns. Priests in both schools had received prior training on child protection and both had established a child protection committee, that aimed to promote awareness of child safeguarding, and offer guidance, counselling and reporting avenues. The urban school was affiliated with a Catholic congregation that had implemented further activities for child protection, including widespread awareness-raising; the inclusion of school counsellors; and development of referral policies. The rural school had not undergone this additional work.

### Purpose of the study

This research is part of an ongoing collaboration between the Zimbabwe Catholic Bishops Conference [ZCBC], London School of Hygiene and Tropical Medicine [LSHTM], Academic Research Centre [ARC], Q Partnership [Q], Childline Zimbabwe [CLZ] and Porticus, with the aim of developing and testing a ZCBC intervention to prevent violence against children in Catholic primary schools. The ZCBC intervention will be implemented in all Catholic primary schools in the country, and aims to promote child safeguarding in schools, and specifically to reduce bullying, corporal punishment and promote a culture of positive values around child safeguarding. The research was conducted as part of formative research to support the development of the intervention. The aims of this paper are to examine how adult school stakeholders conceptualise and enact child protection in schools, and to consider this in light of the challenging structural contexts surrounding schools.

### Sampling and data collection

Key informant interviews (N = 18) were conducted with education professionals and school stakeholders in and around two Catholic primary schools, with this paper drawing mostly on interviews with school-level stakeholders, due to the focus of this study (N = 12). This included 4 parents (2f/2m); 4 teachers (2f/2m); 2 school priests (2m); and 2 headteachers (2f). High-level stakeholders external to the schools, whose views contributed less to this analysis (N = 6), were selected for their key role in child protection and/or education in the local area. These included two local government education actors (2m); one Catholic education actor at diocesan level (1f); two religious actors working at a high level within two different Catholic Orders (1f/1m); and one NGO actor with child protection expertise in Zimbabwe (1m). Initially we aimed to engage children, but due to Covid-19 pandemic restrictions we delayed such engagement.

All interviews were conducted by qualitative researchers trained and experienced working within this context, led by the lead researcher RN (referred to as ‘the qualitative research team’). High-level stakeholder participants were identified through ZCBC and selected for their experience in child protection and/or for their anticipated role in the ZCBC intervention. School-level participants were selected both for relevance to study aims, and for safe accessibility during the Covid-19 pandemic. For practical reasons, we also selected schools that were within a day’s driving distance of Harare and where school stakeholders spoke standard Shona. School headteachers were asked to identify teachers and parents willing and able to participate. ZCBC actors made the initial approach for preliminary consent from high-level stakeholders, and from headteachers for all school actors. Follow-up contact was then made and informed consent sought from all individuals for their participation.

Interviews were semi-structured to allow for a range of views and experiences while also addressing research aims. Interview guides included questions on understandings of child protection; forms of abuse and other protection concerns; important areas for the ZCBC intervention; views on corporal punishment and alternative discipline; and additional questions for higher-level stakeholders according to expertise. Interview guides were included in both English and Shona so that participants could choose their preferred language. The qualitative research team collaboratively translated key concepts to ensure accuracy and relevance for the setting, using contemporary Shona terms that are colloquially used. For example, child protection was translated as ‘kuchengetedzwa kwevana’, meaning the literal care and protection of children. Any disputes in translation were resolved by the lead researcher (RN), using the terms most commonly agreed upon among the team. Stakeholders were asked their views on child protection policies generally in schools. All school staff and parent interviews were held in schools. Participants were offered the choice of conducting the interview in Shona or English. Most school-level stakeholders chose predominantly Shona and most high-level stakeholders chose predominantly English, however interviews combined both Shona and English as is customary.

### Data analysis

The qualitative research team transcribed all interviews into English, and translated them during transcription where necessary. These translations were checked for quality by a senior qualitative researcher and translator. Transcripts were analysed in English. Analysis was thematic, in order to identify themes that emerged across the data as a whole [49], and also involved some techniques from grounded theory approaches, such as in vivo coding and constant comparison, as it was important for this study to remain closely rooted in the data and seek meaning in participants’ own words [50, 51]. We employed both inductive and deductive coding, as we were interested in clear concepts that were of relevance for the study, and yet we also sought to respond to emergent themes. Analysis was led by ET and RN and involved interpretive input from the broader study team, comprising academics from the fields of education and public health, including social scientists working within Zimbabwe on education, child protection and economic behaviours; and education professionals and practitioners with expertise in international child protection and Catholic education in Zimbabwe. An initial code list was drawn up prior to the research, then emergent themes were explored following data collection through a range of techniques, including repetition and ‘missing’ data [52], wherein we identified key thematic areas that did not emerge in the data, but we knew to be relevant in this context [52]. Analysis involved several rounds of coding and reorganisation of themes: First the qualitative research team conducted coding across the data, led by RN and ET, using and revising the initial code list; second, ET and RN further refined the code list and conducted a second round of coding and identification of themes; and finally, the themes were shared with the broader study team for input, and further refined. These steps were iterative. All data was coded using NVIVO 12 software [53] by ET and RN and the qualitative research team who had undergone training in NVIVO. This facilitated triangulation and reflection on our selection of data and themes.

### Ethical considerations

Ethical approval was obtained from the Medical Research Council of Zimbabwe and LSHTM. Due to Covid-19 challenges, all tools and consent procedures were amended to have remote options and researchers underwent training in remote methods. Only two interviews with high-level stakeholders were held remotely. All participants gave written informed consent for face-to-face interviews while for remote interviews, consent was offered verbally and recorded in written format by the researcher. Researchers received nine days training on violence research, which included sensitive interview techniques; identifying signs of distress; scripted responses for handling disclosures of abuse; and steps for follow-up action. A safeguarding referral mechanism was developed in collaboration with Childline Zimbabwe [CLZ], and includes a referral structure for violence disclosures, including level of severity and algorithm for response action. During this formative phase of the research, no disclosures were made that warranted referral, although one case was followed-up through discussion with the study team, as per the protocol.

## Findings

### Conceptualisations and perceptions of child protection

While national policy views child protection in terms of formal ‘services and mechanisms’ and also focuses explicitly on violence, abuse, exploitation and neglect, the adult school-level participants in our study conceived of child protection in broader and more informal terms. When asked how they understood child protection and what aspects were important to them, teachers, parents and priests viewed child protection largely as broad support for children’s wellbeing and development. This approach, which we refer to as ‘holistic support’, included overall provision and guidance; discipline, monitoring and behaviour management for children’s conduct; and training for children’s self-sufficiency to protect themselves from harm. This overall concept of child protection focused on the role of adults in caring for children, and not often explicitly on child gender.

#### Child protection as holistic support

Adult school stakeholders most significantly viewed risks to children’s protection in terms of the generalised threat of unsafe environments and unknown adults. In response to these perceived risks, they conceptualised child protection as support important for children’s safety and development in a risky environment, including material and financial provision; discipline for children’s good conduct and behaviour, and prevention of peer violence; limiting children’s exposure to risky situations; and adults responding to abuse:

What is your understanding of child protection?

Child protection involves safeguarding children like for example here in the rural areas, children should not walk alone […]

Do you think child protection policies are necessary in the school?

Yes, they are necessary. The policies at school help because, for example, when they get to school they are kept in the school premises… it’s nice to keep them in the yard because a lot of mischief happens, that is when you hear issues of some breaking limbs and some are fighting […]

What areas of child protection are important to you?

There is a food aid programme here, so they are supplied with maize or mealie meal. [Parents] contribute money depending on the number of children one has at the school… it is good for everyone to contribute

Female parent (#1), rural area

Here child protection is viewed as protecting children from environmental dangers, managing children’s behaviour, and providing food and money. Holistic views of child protection emerged often:

Child protection means we are protecting the child from any physical and emotional harm. We want the learning environment to be free from any physical violence or emotional violence

[…]

Okay, anything else you want to share with me with regards to child protection policies at this school?

Yes, the other one is that we want to mould a child emotionally, intellectually and spiritually. We always have church masses when we open school, during the term and just before we close schools because we want to build a child holistically. We also encourage children not to be a danger to themselves. We try always to make sure there is no bullying by telling learners to open up

Male teacher (#2), urban area

This teacher views protection as creating a safe environment, while also emphasising the importance of ‘moulding’ a child ‘holistically’. There is a focus on children’s behaviour and peer violence, seen here with ‘bullying’ and above with reference to ‘mischief’, ‘breaking limbs’ and ‘fighting’. The Catholic Church played a role in such conceptualisations of protection, with religious teachings seen to offer moral guidance for children. Interestingly, teacher violence was not discussed as a key risk to children’s protection, and violence from caregivers was not often discussed. Overall, participants viewed generalised community dangers; health and safety risks associated with poverty; violence from unknown (or lesser known) adults, including particularly sexual violence for girls; and peer violence as the biggest risks to children, and saw child protection as holistic support to mitigate these risks.

#### Child protection as discipline and behaviour management

While child protection was viewed in holistic terms, the most commonly agreed upon and emphasised aspect was maintaining children’s good behaviour, for both boys and girls. Child protection was most often seen as guidance required for children to protect themselves and uphold good conduct, and protection was frequently viewed as synonymous with discipline:

What is your understanding of child protection?

Child protection is that these children need to be guided at home, school, community, so that that they live happily and safely

[…]

Do you think child protection policies are necessary in this school?

They are very necessary because there are children with issues who need to be assisted. If they lack that support, they may go wild

Female teacher (#3), urban area

Teachers could often accompany a general focus on children living ‘happily and safely’, to an explicit focus on behaviour, as here children lacking protection are described to ‘go wild’. The school was seen as key for this form of child protection, with guidance and behaviour management viewed as essential functions of the school. Parents tended to view child protection in schools as a form of teacher-led behaviour management:

Do you think child protection policies are necessary in the school?

As a parent I say, yes, they are necessary

Why?

The policies help mould children into well-mannered human beings, children who listen to their teachers and respect parents. When children behave in such a manner then parents will know that their children are getting the best from school

[…]

[Schools opening] is a relief to parents because it also means children’s mischief is coming to an end, as they will be spending more time with teachers and we know they will be protected

Male parent (#4), urban area

An emphasis on peer violence as a risk to children often led to a focus on protecting children from one another, and an alignment of protection with behaviour management:

Do you think child protection policies are important in the school?

Yes, they are very essential

Why?

They can be abused even in the school environment because during play time [children] can bully others, [they] can fight, they have sexual activities at school. So it is good to have them because it guards against bad conduct

Female teacher (#5), rural area

The focus on discipline and behaviour management as a form of protection was therefore twofold: Firstly, participants emphasised the need for discipline as protection and to guide children to live safely. Secondly, viewing peer violence as a primary risk to children led to strengthened emphasis on discipline as protection of other children.

#### Child protection as creation of self-sufficient children with responsibility to protect themselves

The close relation between protection and discipline also related to a tendency for adults to place responsibility on children for their own protection, which was at times gendered. Adults often expressed concern at community risks and viewed children as active in negotiating their environments safely, and responsible for doing so. In these moments, protection entailed guiding children to make good choices and avoid harm:

As we all know, a lot happens in this community, there are a lot of businesses and if we do not protect our children, they may start picking plastic containers and copper wire for resale. Our duty as parents therefore is to protect our children so that they do not grab anything and everything that presents itself to them

Male parent (#4), urban area

A local government education actor described his views on what a child protection programme should involve:

A child protection programme at school should aim to create a learner who can take care of himself who should be free to operate under any [situation]. That kind of learner is the child that we want to create. Someone who is self-sufficient in terms of creativity, someone who should be empowered in terms of decision-making […]

So how do you ensure that that person is empowered, how does it happen?

Usually we would want to share information with our learners… If we consider topical issues like, HIV & Aids […] the learner should be [able] to make a decision that would make sure she is not infected or affected by the [HIV/Aids] pandemic. In other words, I just singled out a particular point where we consider that our learner is empowered, the learner should be able to decide if ever she is going to indulge, then safety should be considered as key in whatever the learner does

Local government education actor (#6), rural area

This narrative of child protection focuses on learners’ abilities to take care of themselves, framed as self-sufficiency and empowerment. In such a framing, the role of perpetrators of abuse, or adult caregivers to protect children, is downplayed, with high responsibility placed on children. As the switch in pronouns from ‘himself’ to ‘she’ suggests, there was a particular emphasis on girls. Girls were widely seen to be at additional risk of sexual exploitation and needing protection, while at the same time presented as active in choosing or refuting such exploitation:

Do you think child protection policies are necessary in this school?

Yes, they are necessary

Why do you say so?

Because if learners go untaught maybe they will indulge in something that is not good, not because they like it but also because they don’t know how bad it is

[…]

The world they are living in is now bad. Even the elders they tend to tamper or to play around with those little kids maybe because of hunger or something. When a child or maybe a girl child starts to meet a sugar daddy she may be tempted because of hunger, so they need to be guided always

Male teacher (#7), rural area

The risks presented here relate to the environment, poverty, and older perpetrators, and protection is viewed as teaching and guiding (female) learners. Here with ‘sugar daddy’, the teacher focuses on girls’ relationships with older men and transactional sexual relationships. While the responsibility of adult perpetrators of violence and exploitation is acknowledged, with ‘elders’ ‘[tampering] around’, ultimately the responsibility is placed on girls to resist ‘[temptation]’ and protect themselves.

In such conceptualisations, which featured across the data, the primary focus of child protection was for adults to guide and create children who were well-behaved, self-sufficient and ‘empowered’ to protect themselves from abuse. This perspective was applied to both boys and girls, although was particularly marked for girls amid discussion of sexual violence and exploitation and a perception that girls would be able, and should, ‘resist’ temptation towards exploitation and protect themselves from harm.

### Responses and reactions to child protection concerns

Adults described a range of different responses in practice, and personal reactions and feelings, to child protection concerns. When asked about how cases of abuse or their child protection concerns were handled in schools, school staff described referring some cases through formal mechanisms, such as reporting to the police for sexual abuse, while for some other cases, such as teacher physical violence, formal referral was not mentioned. School staff and parents also described a range of personal reactions and feelings to child protection concerns, including burnout and demoralisation; and, in some cases, blaming or stigmatising children.

#### Responding to child protection concerns through formal mechanisms

School staff in particular described referring certain child protection concerns through formal mechanisms. Reporting to the police was the most discussed action for cases of child marriage or severe sexual assault, such as rape in the family or community. While these discussions emerged in both schools, staff from the urban school described supportive school structures and actors more often than in the rural school, possibly due to its increased child protection training:

We have had some cases that had to go to court. There are people who have been convicted and are in prison now. Men who have abused girls, some of our girls, outside the school but linked the school because they are our students. Our school counsellor had to follow a number of these. In the last two years there has been one conviction and someone is in prison

School priest (#8), urban area

While all participants agreed that cases of child marriage and child rape should be referred to the police, handling these cases were not always described to be straightforward in practice, with formal referrals facing a range of challenges. One priest in the rural school described challenges around reporting high-status perpetrators through village child protection structures:

There might be some untouchables in the community or in the family, you can’t report your own, you can’t report those from the chief’s family (royalty). The way cases are treated, if it’s at a village court they might not treat the cases sufficiently and professionally, they might not help the child

School priest (#9), rural area

In the urban setting, one teacher described challenges with court processes as she attempted to support a learner through a disclosure of familial sexual abuse:

I think the main worry is that when a child is being abused, usually nothing will come out due to corruption. I remember one time there was a child who was abused… the teacher asked me to talk to the child, so I became friendly to the child, we talked about it time and again, until she revealed that she was sexually abused […] We went through child protection and justice, and the case was held at X. Nothing came out of this

[…]

When we went to court… they started laughing and the child was watching. The child asked me ‘ma’am why are they laughing?’… I didn’t want her to notice so I told her they were laughing at their own stories. They asked me if I could continue coming, then I said I could not because I had a class with 45 children, I couldn’t keep coming there. Then they asked about the bus fare, and I said I did not have money, then they started laughing

Female teacher (#3), urban area

Several challenges can be noted in this teacher’s experience: First, as in the priest’s example from the rural case, she describes the fear that cases will not be resolved due to ‘corruption’; Second, the description of court structures, such as the court officials’ laughter, suggests the case was not taken seriously; Third, her description of personal challenges managing the bus fare and her large class size, suggests her handling of this case was individualised, and points to a notable absence of broader, structured institutional support from the school or other formal structures.

In addition, lack of resources and additional challenges amid the Covid-19 pandemic were seen as a major issue for child protection at an institutional level. The priest in the urban school described the following:

It should not be an excuse but at times you are dealing with issues that drown you even in this Covid-19 reality. Our safeguarding alert lights were almost dimming because there are things that we are really trying to push […] It’s always bound to suffer when you have pandemics such as Covid-19, but I think the issue is to get the child safeguarding topic as a bread-and-butter issue, so that even when things become tough it remains part of the constrained budget

School priest (#8), urban area

Here the priest refers to a ‘dimming’ of ‘safeguarding alert lights’ to describe a generalised lack of focus on and prioritisation of child safeguarding concerns in relation to the challenges of the Covid-19 pandemic.

While some cases, such as child rape and child marriage, were seen as warranting formal referral, therefore, such referrals often faced challenges in practice. In addition, it was striking that certain child protection risks were absent in discussions of formal structures. Pregnancy among female pupils and caregiver physical or emotional abuse were rarely mentioned as warranting referral, or were described to be handled outside the school. Examples of teacher physical, sexual or emotional abuse of learners were not discussed by school staff or parents when asked about child protection. There was, therefore, a clear distinction between what was, and was not, described to be handled through formal child protection mechanisms, with a particular absence around teacher-perpetrated abuse. For those cases that were referred, adults emphasised the challenges they faced in doing so.

#### Reactions to child protection concerns

In addition to formal referral action, teachers discussed a range of personal reactions to child protection concerns. These included feeling burnt out and demoralised, as well as perceptions of children’s own culpability. Burnout and demoralisation was particularly described by those teachers who had attempted to engage in child protection referrals, and had faced challenges in doing so. School staff all recognised the huge burden placed on teachers amid the significant and protracted challenges they faced in their professional and personal lives, leading to feelings of demoralisation and hopelessness:

Are there any forms of abuse that teachers or adults face in this community?

Emotional abuse. Teachers no longer have money and so they have lost that kind of respect from the community as everyone says teachers don’t earn much, teachers are poor

How does this make you feel as a teacher?

I usually think of quitting […] teachers are abused [teased] for their very poor remuneration

Male teacher (#7), rural area

Such challenges were viewed as significantly detrimental for child protection, which could be viewed as an additional pressure for already overburdened schools and staff:

Our teachers are quite distraught with the employer, that is the government. So levels of motivation, enthusiasm and energy are at their lowest because they are earning [too] little to be able to sustain their families. These are the key people who are taking care and teaching the kids […] We are struggling with trying to keep our teachers motivated and a bit more effort needed on child safeguarding may come as an extra burden

School priest (#8), urban area

Because these teachers are incapacitated… already they are being bombarded with a lot of work without pay. Just being told of child protection, they will think it is extra work so they won’t listen

Female teacher (#3), urban area

The female teacher cited here, and who above described her experience of supporting a child through the courts, felt distressed by the lack of resolution for the case:

I went there in an effort to help the child, but you can see that there is corruption in the world. As much as we try to help these children, as a child protection person it will end up being part of you. Because, as you are going through the process with the child, you will be promising them that maybe something positive will come out of it. But in the end… […] So I had promised her that her life was going to be better in the future…I told her that she was going to be someone in life and that we would fight for her rights. But you will see that there is there is no real end to the story

Female teacher (#3), urban area

The sense of demoralisation and futility that the teacher feels is evident. She describes her desire to help the child, and the practical and emotional challenges she faced in doing so. Elsewhere in the interview, this teacher explained that encountering such issues could leave teachers feeling overwhelmed and lead them to avoid supporting children:

You can’t even handle them or come to understand them, because if you try to ask you will hear stories here and there, then you say ‘ahh why can’t I just move away from those issues and leave them alone…’

What are the significant challenges that teachers in this school face when working with child protection issues?

Some of the challenges which I think are being faced is that some of the children need to be protected, but some of the teachers they will be having their issues as well which won’t be addressed. So, addressing this case when your case is not addressed will be another issue

Female teacher (#3), urban area

Feelings of burnout amid the challenges teachers face in responding to abuse, the lack of resolution that this teacher experienced, and the fact that teachers face their own challenges and receive little support, were perceived to affect teachers’ engagements in child protection.

When discussing cases of abuse or child protection, adults could also respond in ways that were stigmatising, or that held children responsible. Such stigmatisation occurred around perceived ‘errant’ behaviours, and particularly in relation to girls’ experiences of sexual exploitation and adolescent sexuality and pregnancy. All participants acknowledged that children faced structural and environmental challenges during Covid-19 lockdowns, however when describing children presenting at school with child protection issues, they also often focused on perceptions of children’s poor conduct. This occurred across the data, however was more common in the rural setting:

Lockdown might also have caused some economic challenges in some families and in those families, children might have exposed themselves to abuse for survival. When it changed the system and programme of learning, in idleness the children might have started indulging in destructive and abusive lifestyles because they were idle, not going to school and not doing anything, having overstayed at home they are now exposing themselves to abuse

School priest (#9), rural area

Language here such as children ‘exposing themselves to abuse’ resonated across participants’ discussions. Risks to children were portrayed as emerging both from the environment, and from children’s poor conduct. This tendency was particularly evident when related to sexual behaviours among girls, as stigmatisation for cases of pregnant learners could at times be striking:

Impregnating is not due to rape but consensual sex [laughs] I would say Covid-19 has provided an opportune moment for children to be off school having nothing to do with school. They will be playing and end up engaging in ill behaviours like indulging in sex, alcohol and drug abuse

Male parent (#10), rural school

Have [Covid-19 lockdowns] changed the nature of your role in any way?

It has changed a lot the nature of my role because the learners we used to meet with have completely lost control over themselves because most of them became parents

How is that so?

Because some learners became impregnated

Male teacher (#7), rural area

Here participants locate blame on children, their choices, and a perceived inability to control themselves. While there is stigmatisation for a perceived ‘idleness’, ‘destructive and abusive lifestyles’, and ‘sex, alcohol and drug abuse’, in ways that applied to both boys and girls, the emphasis on pregnancy points to a particular focus on girls’ sexual conduct and shame of pregnancy.

Girls could also at times be portrayed as being manipulative or sneaky with regards to sexual behaviours, and this particularly emerged in conversations with adult women around management of girls’ behaviour. In some cases, adults discussed how girls were prone to lying about their behaviour, which in the context had sexual connotations:

Some [learners] will come to you because some are close to the teacher, they will tell you, ‘my mother beats me.’ Then I say, there is no mother who will just enter the house trying to beat you for nothing… The only way I can help you is calling your parent so that we hear what you…’no, no, no, don’t call, you will start something for me’ […]

You will see from her character that her character is changing, maybe the mother will be trying to mould her, but then it will be too late. Then the mother will be frustrated saying, ‘I am out there working for you, and then you are doing something…’ which she did not even mention to me she had done. So, you see that is the problem with these children

Female teacher (#3), urban area

Here the teacher blames the child for experiencing physical abuse from her mother, perceived as a necessary form of discipline to manage the girl’s conduct. Such focus on blaming children either for ‘exposing’ themselves to community abuse, or for experiencing physical punishment as a sign of their poor behaviour, occurred often, and had particular emphasis when relating to girls’ sexuality.

A female parent in the rural area described her perception that girls were ‘naughty’:

Are there any other children that might be at added risk of abuse?

The girl child is the most naughty, they can lie that they were asked to come to school for lessons over the weekend

Female parent (#1), rural area

Here again, the description of girls lying had sexual undertones in this context, and this links a question around ‘abuse’ to a sense that girls were sneaky or manipulative about their (sexual) behaviours.

Interestingly, the same female teacher cited in the first quotation here (teacher #3), described her feelings of demoralisation and desire to help a female learner with a disclosure of sexual abuse (described above), suggesting a complex engagement with responding to abuse. This teacher may have drawn a clear distinction between what she saw as sexual abuse and her perception here of the girl’s misconduct. In a more complex reading, considering the close association of discipline and protection, it is also possible that disciplining learners in this way was seen as an essential part of child protection. Stigmatising and blaming children for abuse, may therefore have been closely interconnected with desires to protect and safeguard them, and the discipline relied on to do this.

While participants across the data could engage in stigmatising narratives around children’s experiences of abuse, there were also some differences. Adults in the rural areas were more stigmatising of children than those in the urban area, who also tended to focus on children’s vulnerability to risks in the urban environment. While the two women above described frustration, blame and stigmatisation of girls’ (sexual) conduct from the position of their involvement in trying to control and manage it, men more often positioned themselves as further from girls’ behaviour and engaged in more detached notions of stigmatisation.

In addition to, or at times instead of, describing how cases could be referred through formal mechanisms, or their own feelings of demoralisation, school staff could therefore often stigmatise or blame children who presented at school with child protection issues. Interlinked with the high level of responsibility placed on children to protect themselves, teachers’ responses to children experiencing abuse or other forms of risk, could thus involve stigmatisation for their perceived poor conduct, and this could be gendered in nature.

## Discussion

This study aimed to examine how adult school stakeholders conceptualise and enact child protection in and around two Catholic schools in Zimbabwe, within a context facing structural, environmental, and resource-related challenges. Five key findings emerged. First, in contrast to national policy that emphasises formal mechanisms to prevent and respond to abuse, child protection was largely conceptualised by participants as holistic support for children’s wellbeing and development. This included a particular focus on children’s behaviour and responsibility to protect themselves from harm. Second, there was a tendency for adults to stigmatise and blame children experiencing abuse or otherwise at risk. This was particularly significant for girls’ sexual behaviours and experiences of exploitation and pregnancy. Third, formal child protection mechanisms were discussed predominantly in relation to child marriage and child rape in homes and communities. Participants largely did not discuss teacher-perpetrated abuse as a child protection risk that warranted formal referral. In addition, staff handling referrals faced many challenges amid a notable absence of institutional or structural support. Finally, teachers in this study faced a range of personal challenges and felt demoralised and overburdened. Our findings suggest that this influenced their engagements in child protection.

The finding that, in contrast to national policy, school stakeholders’ perceptions of child protection were holistic in nature and centred around guidance and discipline of children, resonates with previous findings from Zimbabwe and other sub-Saharan African settings. Some existing studies in sub-Saharan Africa highlight how discipline can be seen by adults as an important aspect of childrearing and protection [54, 55], and care within the context of HIV and AIDS [7], and thus constitutes a disjuncture from international policy emphases. In this challenging context in Zimbabwe, amid patchy formal mechanisms, the adults in our study may have focused on children’s conduct and self-sufficiency as a pragmatic choice about how best to keep children safe. Previous findings from Zimbabwe have also shown that teachers’ feelings of demoralisation and experiencing their own unresolved difficulties, can lead to resentment of children, burnout and feelings of being unable to support children with their problems [7, 9]. Our findings here that adults could stigmatise and hold children responsible for abuse, suggest that in light of the challenges that teachers faced and the lack of support or adequate remuneration they received, teachers may have felt resentful and critical of children, at times extending a focus on their conduct from one of protection, to one of blame. While focusing on children’s behaviour and responsibility was seen as essential for their protection, it could also therefore, underpin stigmatisation of children within this challenging context.

This finding also had gender implications. While our sample size was small, there were some indications that adult women’s stigmatisation of girls’ behaviours may have indeed been closely linked to their efforts at managing girls’ (sexual) conduct and behaviour, and protection from harm. This resonates with other qualitative research in this context showing that adult women in Zimbabwe, particularly caregivers, are key actors for girls’ protection from sexual violence, but that in so doing, they can also be involved in regulating girls’ sexual conduct (Turner et al., forthcoming). Further, a tendency to blame and stigmatise particularly girls, also speaks to studies conducted in other sub-Saharan African [56, 57], and Zimbabwean [47, 58] (Turner et al., forthcoming) settings, suggesting that ‘protection’ can be linked to control and stigmatisation of girls’ adolescent sexuality. The fact that child sexual abuse (of girls) was seen most unequivocally as a child protection case warranting referral, but simultaneously girls’ sexual exploitation was highly stigmatised, suggests an interesting interplay between social norms and policy. We also note a particular absence around the sexual abuse of boys, which we know occurs in Zimbabwe [40], yet qualitative research suggests is shrouded in gendered taboos and stigma [46]. Further research employing a gender lens is important to examine these areas. There was evidence to suggest that affiliation with the Catholic Church provided schools with meaningful support, and research to examine the relationship between religion and child protection, as examined elsewhere [54], is needed in this Zimbabwean context.

The findings that child marriage and child rape tended to be viewed as child protection cases warranting formal referral, but physical violence from teachers and caregivers, and teacher violence in general, were not, are perhaps unsurprising given the policy context. Zimbabwean law requires mandatory reporting of child sexual abuse. Physical punishment of children was legal in schools until only several months prior to the research, is common [40], and physical punishment in homes is still permitted and upheld by longstanding social norms [42, 43]. These findings also resonate with recent analyses of helpline data for referring violence in Zimbabwe, where sexual violence was considerably more likely to be reported than physical violence in general in the country, and teacher violence in general was unlikely to be reported (anonymous, forthcoming). These findings suggest that recent policy shifts preventing teacher physical violence, and the lack of stipulated repercussions for perpetrators, have been as yet insufficient in encouraging teachers to discuss it, and likely conceptualise it, as a child protection issue that requires formal response in schools.

Additionally, our research found that when teachers did refer child protection cases formally, they faced a range of challenges. Existing research emphasises that enacting policy on violence in schools is challenging where doing so contradicts gender and childrearing norms, and requires multi-sectoral collaboration and between actors at different levels [27, 59]. Previous studies also found, as we did, that within weak policy frameworks, teachers responding to child protection concerns often do so informally and in individualised ways, and can be left overburdened and exposed without adequate institutional support, leading to feelings of overwhelm and burnout [9, 19]. Our findings support this, offering further evidence from this context of teachers’ incapacitation and decreasing salaries; the Covid-19 pandemic; and other multi-faceted challenges. Some existing evidence suggests that for cases of sexual violence this particularly relates to female teachers [19], as we found in the case of one teacher in this study. Further research with a larger sample size would be needed to unpack the gendered significance of male and female teachers’ involvements in child protection.

Our study has important strengths and limitations. Firstly, we share new findings on an underexplored topic, examining how teachers conceptualise child protection in challenging circumstances while also employing a focus that takes account of their role as potential perpetrators of violence. Due to our qualitative methodology, our findings should not be interpreted as generalisable to other contexts. We had originally planned to conduct a larger number of interviews and participatory workshops, and to include children’s perspectives. Unfortunately, Covid-19 related restrictions precluded this, and importantly, the absence of children’s accounts here mean we have only partial information on conceptualisations of child protection in schools. The small sample size may also mean that further possible themes were missed. Our analysis benefited from the range of interdisciplinary and international experience within the study team, however. In relation to our finding that teachers did not discuss teacher-perpetrated abuse as a child protection issue, it is also possible that they did not feel comfortable discussing this with us. A longer period of data collection may have supported relationships to explore this further. This absence is an important finding, however, for highlighting the distinction between what was, and was not, openly considered a child protection concern in these Zimbabwean schools.

There are several implications for policy, practice and future research. Firstly, teachers and schools require greater support to be able to operationalise policy frameworks around the protection of children in Zimbabwe. Further research is needed to understand how recent policy shifts preventing physical punishment have been communicated to and understood by school stakeholders, and there is a need for development of policy that clearly stipulates repercussions for teacher perpetrators of abuse. Interventions with school staff and parents, addressing social norms around teacher physical punishment and examining its positioning as a child protection concern, are also needed. It is also important to understand how interventions can strengthen response pathways for violence in schools. In contrast to primary prevention interventions to reduce teacher and peer violence, to our knowledge, no school-based intervention to identify and refer child protection cases to formal structures has yet been rigorously evaluated, and evidence is lacking. Qualitative evidence into a school violence response mechanism in Ethiopia [60] found that teachers were well-positioned actors, but responding to abuse could leave them overburdened and vulnerable to repercussions. Further research into interventions that meaningfully support teachers responding to abuse, and that create effective mechanisms for doing so formally, are needed. Children’s perspectives are needed at the heart of this research, to understand how they conceptualise their own protection, areas of importance, and their views on effective disclosure and referral mechanisms. The findings of this study will feed into further research, taking these concerns forward, and aimed at supporting the refinement of this intervention to address and respond to violence in Catholic primary schools in Zimbabwe.

Our findings on teachers’ demoralisation suggest that any intervention seeking to involve teachers in child protection needs to take account of the challenges teachers face in particular contexts. To be effective, interventions will also likely need to be viewed by staff as offering support, rather than additional pressures, and avoid disempowering teachers through confrontational approaches, as was important in interventions in Jamaica and Uganda [61, 62]. It may also be helpful to offer teachers pastoral support. At the same time, there is also a clear need for interventions to address the tendency in this setting to hold children accountable for abuse, alongside approaches that address unequal gender norms that underpin blame for girls experiencing sexual exploitation and abuse. Blame and stigmatisation leaves children unsupported, strengthens inequalities and, further, underpins children’s reluctance to come forward [15, 63, 64]. Violence in schools, and responses to it, are closely related to gender norms and inequalities, and require gender-sensitive policy and intervention approaches [27, 59]. Child protection interventions in this context should address child-blame and shift responsibility from children to adult perpetrators, and construct supportive environments for children experiencing abuse, alongside further research into how this can also address unequal gender norms.

## Conclusion

Adult school stakeholders facing a range of challenges in Zimbabwe, conceptualised child protection as holistic care for children with a focus on discipline, children’s behaviour and their responsibility to protect themselves. Adults did not largely discuss teacher-perpetrated abuse as a child protection concern, and predominantly focused on community dangers, particularly child marriage and child rape. Responding to child protection concerns in schools was challenging for teachers and there was a lack of clear institutional or structural support, leaving teachers feeling demoralised, burnt out and lacking in motivation for child protection. There was also a tendency for adults to blame and stigmatise children, which was gendered in nature. There is a need for child protection policy and interventions to address teacher-perpetrated abuse, particularly physical punishment; to shift blame from children to perpetrators of abuse and address unequal gender norms; and to offer meaningful support to teachers living and working in challenging circumstances.

## Supporting information

S1 Questionnaire (DOCX)
